# Advances in Combined Drying Techniques for Enhancing Dried Fruits’ Quality, Drying Efficiency, and Energy Conservation: A Critical Review

**DOI:** 10.3390/foods15173019

**Published:** 2026-08-27

**Authors:** Nonhlanhla Vezi, Yardjouma Silue, Olaniyi Fawole

**Affiliations:** 1 Postharvest Agroprocessing Research Centre, Department of Botany and Plant Biotechnology, University of Johannesburg, Auckland Park, Johannesburg 2006, South Africa; nonhlanhla94vezi@gmail.com (N.V.); siluey@uj.ac.za (Y.S.); 2 South African Research Chairs Initiative in Sustainable Preservation and Agro-Processing Research, Faculty of Science, University of Johannesburg, Auckland Park, Johannesburg 2006, South Africa

**Keywords:** combined drying techniques, bibliometric analysis, fruit quality, energy consumption, drying efficiency

## Abstract

Fruits are widely consumed for their rich array of vitamins, minerals, and health-promoting compounds. However, their high perishability due to elevated moisture content often results in significant postharvest losses. To tackle this global challenge, drying represents one of the oldest preservation strategies. In the last decade, advances in drying strategies have led to the emergence of combined drying techniques. This critical review synthesises the existing literature on combined drying techniques for fruit preservation, focusing on publications from the Scopus database. The integration of bibliometric analysis with VOSviewer yielded 67 journal articles and an evaluation of global research trends, geographic contributions, and network visualisation. From a mechanistic perspective, combined drying techniques enhance fruit quality and drying performance while minimising energy consumption and production costs, thereby promoting global sustainability. However, various challenges hamper their broader acceptance, including the high upfront investment, a lack of legislative backing, and technical difficulties that prevent large-scale deployment. This dual approach (bibliometric and systematic review) highlights these challenges and proposes future research directions for combined drying techniques for fruits.

## 1. Introduction

Fruits provide a variety of nutrients beneficial to human health, such as vitamins, minerals, dietary fibres, phenolics, carotenoids, and other bioactive compounds [1]. Balanced diets that include fruits are essential not only for preventing malnutrition but also for boosting the organism’s defence against numerous diseases [2]. Additionally, fruits play a significant role in global economic growth, contributing to household income and, especially, rural employment and economic development [3].

However, fruits incur significant postharvest losses throughout the supply chain due to their high perishability, which is linked to their active postharvest physiology and susceptibility to microbial spoilage. This rapid deterioration, leading to a short shelf life, is primarily associated with their high moisture content (commonly above 80%), high respiration rate, and sensitivity to handling damage [4]. Furthermore, the growing world population heightens the demand for food production, which in turn exacerbates the postharvest losses and food waste [5]. For instance, South Africa alone loses an estimated 4.2 million tonnes of fruit annually, representing about 47% of the country’s food waste and loss [6]. Post-harvest fruit losses have emerged as a major global concern, affecting the global economy, farmers’ income, and, ultimately, challenging the United Nations Sustainable Development Goals (SDGs).

To overcome these issues, minimally processed fruits, such as ready-to-eat, dried, canned, or frozen fruits, have been presented as noteworthy alternatives with various advantages over fresh fruits [7]. Dried fruits can significantly contribute to ensuring food security, particularly in regions where fruit cultivation is challenging and during off-seasons [8]. Undoubtedly, dried fruit presents a longer shelf life than fresh fruit, offering a greater availability and accessibility even during drought seasons [9,10]. Additionally, dried fruits offer various benefits that meet the growing consumer demand for convenient, healthy snacks [11].

While dried fruits offer numerous benefits, the drying process remains critical for balancing quality, drying efficiency, and energy use. Drying reduces food’s water activity, prolonging shelf life by limiting microbial growth and enzymatic activity [12]. Drying is governed by simultaneous heat and mass transfer, in which thermal energy supplied to the product induces the moisture removal from the product to the surrounding environment. The literature presents various drying methods that operate through different moisture-removal regimes, with an initial period dominated by relatively accessible surface or free water, followed by a falling-rate period in which internal moisture diffusion increases, thereby controlling the process. Studies have reported that traditional drying methods, such as sun or air drying, have substantial limitations, such as lengthy procedures, and can negatively impact the quality of the dried product [1,13]. This has led to the development of advanced drying techniques, including hot-air, microwave, vacuum, infrared, freeze-drying, and oven drying [14]. However, these drying procedures have drawbacks, including nutrient loss, discolouration, off-flavour, structural changes, high energy requirements, and high cost [15,16]. Therefore, combining multiple drying techniques has emerged as a promising alternative for achieving a sustainable drying process without compromising fruit quality. These new-generation drying technologies, known as hybrid drying technologies, are increasingly being adopted to address these drawbacks by capturing the synergistic benefits of the involved methods while mitigating the drawbacks of the individual methods [17]. Thus, several studies have shown that combined drying technologies enhance energy efficiency and reduce drying time and energy consumption [18,19,20].

Despite growing interest in combined drying technologies, previous reviews essentially focused on their effects on product quality. However, an integrated understanding of how the research on these new-generation technologies evolved, how complementary drying mechanisms interact, and how they improve drying kinetics, energy efficiency, and dried fruit quality is essential for future developments. Additionally, the distinction between pretreatment-assisted drying and combined drying technologies (i.e., the integration of multiple drying mechanisms) remains poorly reported. Consequently, a more rigorous and extensive literature review is needed to examine combined drying techniques, utilising both systematic and bibliometric analyses. Therefore, the present study integrates bibliometric mapping with a critical assessment of combined drying mechanisms, their drying performance-quality trade-offs, and practical limitations in fruit drying. Through this dual approach, the review aims to (i) highlight recent advancements and emerging research trends, (ii) identify major research themes and patterns of scientific contribution, and (iii) critically evaluate technological performance, identify knowledge gaps, and propose future research directions for industrial translation of combined fruit-drying technologies.

## 2. Literature Search Approach

**Figure 1:**
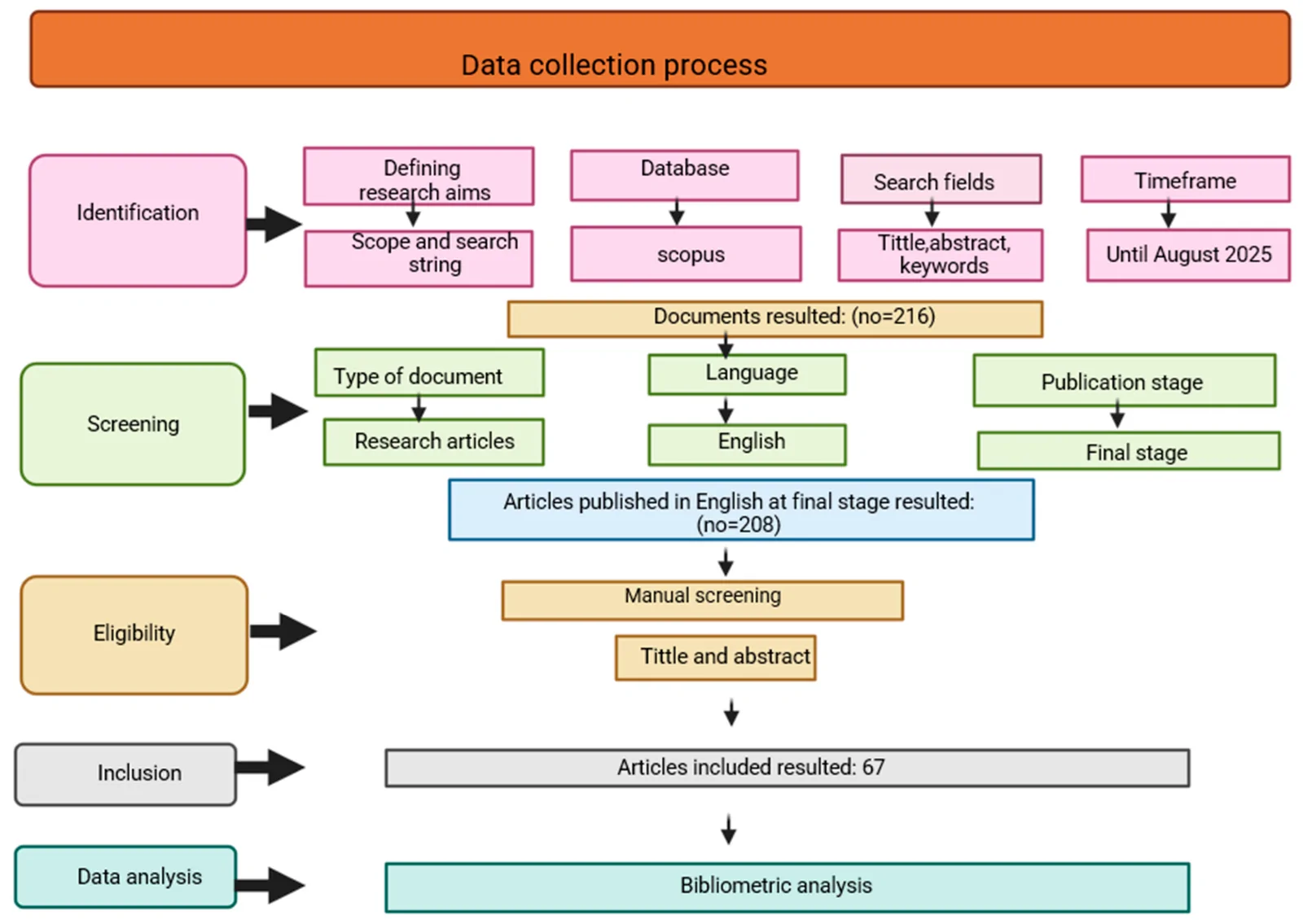
Schematic diagram of data collection.

The data for this study were collected in accordance with the Preferred Reporting Items for Systematic Reviews and Meta-Analyses (PRISMA) guidelines (Figure 1). Four stages, including identification, screening, and inclusion and exclusion, served as the foundation of the PRISMA method [21].

### 2.1. Identification

Data were extracted through the Scopus database on 12 August 2025. We selected the Scopus database for its broader coverage of papers and higher quality compared to other databases [22]. Databases such as ResearchGate and Google Scholar were disregarded due to their poor bibliometric results as reported by [23]. The following search string was used for the identification: (“combined dry*” OR “hybrid dry*” OR “combined dehydration” OR “hybrid dehydration”) AND (“fruit quality” OR “drying performance” OR “drying efficiency” OR “energy consumption” OR “energy savings” OR “energy conservation”). The search strategy was intentionally broad to maximise sensitivity and capture commodity-specific studies (e.g., mango, apple, banana, or pomegranate) that may identify the investigated fruit without using the term “fruit” in the title, abstract, or keywords. As a result, the search yielded 216 documents, including studies focusing on both fruit and non-fruit matrices. Although this approach reduced the specificity at the initial search stage, it minimises the risk of excluding relevant fruit-drying studies. Therefore, the search outcomes were further screened to exclude records that did not meet the predefined eligibility criteria.

### 2.2. Inclusion Criteria and Exclusion Criteria

The inclusion criteria were English-language publications classified as research articles, published before August 2025, in their final publication stage, and discussing the effects of combined or hybrid drying on fruit quality. Studies focusing on a single drying process, on vegetables, or on any other food products except fruit were excluded and could only be used to support mechanistic explanations. Therefore, after the screening of the 216 studies initially recorded, only 67 articles met the predefined selection criteria (fruit-focused original research). Bibliometric analysis was conducted using VOSviewer version 1.6.20. This includes author keyword analysis, annual scientific output, and countries contributing to research development in the field. Figure 1 illustrates the overall process flow for data acquisition.

## 3. Bibliometric Mapping of Research on Combined Drying Technologies

### Global Research Trends and Geographic Contributors in Combined Drying Techniques for Fruit

**Figure 2:**
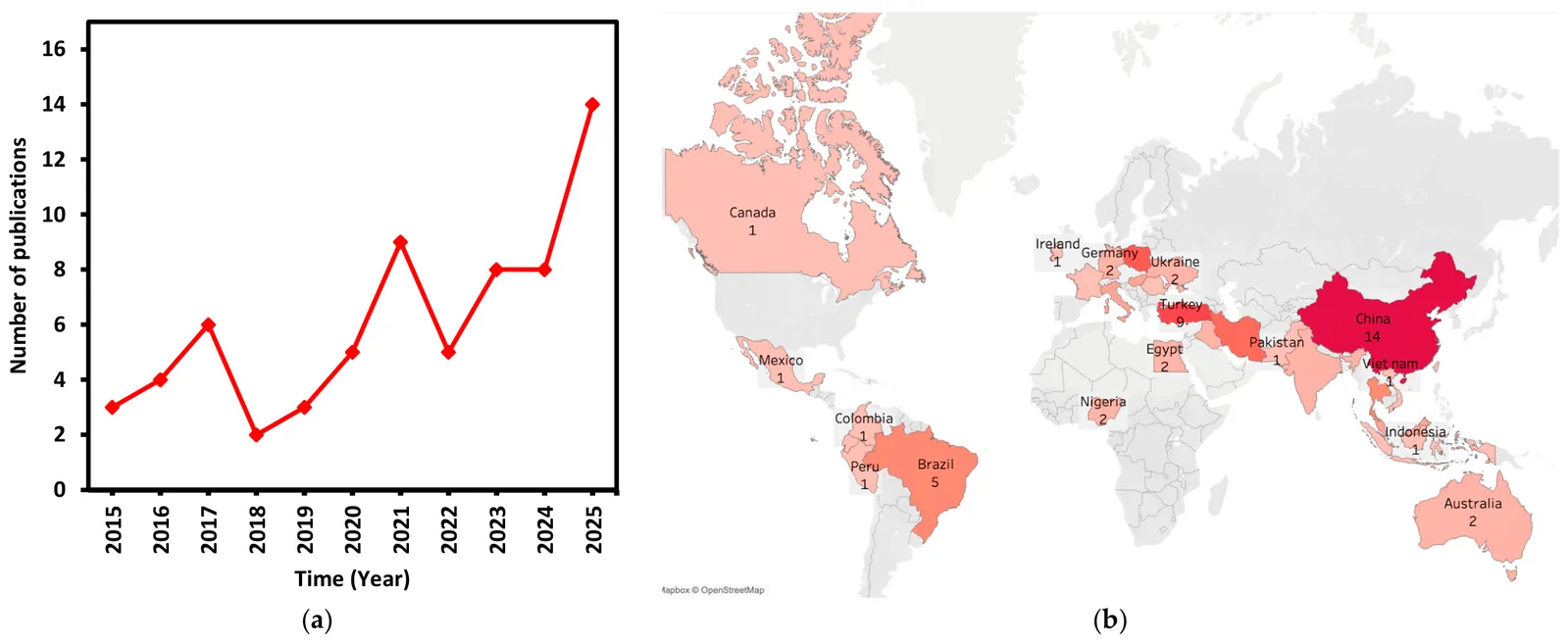
Scientific annual production (**a**) and geographic distribution (**b**) in research on combined drying techniques published in the Scopus database from 2015 to 2025.

Research on combined drying techniques (CDTs) for fruit quality has grown significantly over the past 11 years. Figure 2 shows a growing upward trend in CDT-related publication counts from 2015 to 2025, with intermittent fluctuations. Thus, these findings indicate sustained global growth in combined fruit drying. This may be driven by growing consumer demand for premium-quality products, the need for energy-efficient processing, and a shift in focus towards sustainable food processing. Furthermore, about 27 countries are working to advance combined drying techniques to preserve fruit quality. However, their publication output is highly uneven, with China contributing the most publications, followed by Iran.

Conversely, countries such as Vietnam and Ireland contributed relatively few publications to the field. Overall, the distribution highlights the following strong imbalance: research is predominantly dominated by developed and emerging countries, while developing countries show comparatively low or no participation. However, the bibliometric data alone cannot establish the socioeconomic, institutional, or research-policy factors underlying these findings.

## 4. Overview of Hybrid/Combined Drying Techniques (CDTs)

### 4.1. Definitions

Combined drying is an innovative drying method that applies two or more drying techniques sequentially, mitigating the drawbacks of a single dryer by combining the benefits of each [24]. In addition, a hybrid drying technique integrates two or more drying mechanisms that operate simultaneously as a single, tightly coupled process. These techniques can be operated simultaneously or in a mixed arrangement [25]. Based on this distinction, the different categories and examples of combined and hybrid drying are presented in Table 1.

### 4.2. Fundamental Principles of Combined Drying

A comprehensive understanding of the principles of mass transfer, thermodynamics, process optimisation, and the mechanisms governing structural, physiological, and quality transformations is essential for CDT. This integrative knowledge framework not only demonstrates the complex interactions occurring during the drying process but also facilitates a systematic evaluation of alternative drying techniques. Consequently, it serves as a scientific foundation for the rational selection and optimisation of the most suitable drying technologies to achieve high product quality and improved drying efficiency.

#### 4.2.1. Heat- and Mass-Transfer Principles

In combined drying techniques, different energy combinations synergistically enhance heat and mass transfer. However, the level of heat- and mass-transfer enhancement varies substantially across combined drying systems. Specifically, the combined thermal inputs raise the temperatures of both the surface and the internal layers of the food material by increasing molecular vibrations and promoting faster internal heat transfer [28]. In addition, the temperature rise accelerates heat transfer and moisture migration from the interior of the food material to its surface. In the meantime, the accompanying thermal airflow or secondary heating mechanism facilitates the continuous removal of surface moisture while regulating surface temperature, thereby reducing external mass-transfer resistance. As a result, the integrated thermal actions improve heat and mass transfer and drying efficiency beyond what is achievable with individual dryers [29]. Although thermal–thermal combinations can significantly intensify heat transfer, they may increase the risk of non-uniform heating or localised overheating when energy input is not adequately controlled. Conversely, in nonthermal drying combinations, tissue permeability increases, and microjets disrupt the diffusion boundary layer, forming channels that accelerate moisture migration and evaporation without high temperatures [30]. Indeed, in nonthermal-thermal systems, agitation disrupts structures, enhances diffusion, and accelerates evaporation and internal moisture migration, thereby altering tissue structure through shrinkage [31,32,33]. Consequently, non-thermal combinations primarily enhance mass transfer to limit excessive thermal exposure.

In sum, the selection of a combined drying strategy should consider whether internal moisture diffusion, external mass-transfer resistance, or heat-transfer limitation is the dominant constraint for the specific fruit matrix.

#### 4.2.2. Thermodynamic and Kinetic Principles

In drying technology, modelling transfer phenomena is a useful technique for revealing functional mechanisms, transfer phenomena, and process interaction [34]. Moreover, by using this approach, the distribution of velocity, temperature, moisture, and changes in different physical and chemical properties of food materials, such as porosity, thermal conductivity, diffusion, bulk density, colour, and antioxidant activity, can be examined and predicted using the modelling of transfer phenomena in microwave-assisted drying [35,36].

## 5. Mechanisms of Combined Drying Techniques

### 5.1. Synergistic Mechanisms in Combined Drying Configurations

Sequentially, the mechanism of solar-assisted microwave drying combines low-temperature solar dehydration with rapid, volumetric microwave preheating [37]. Utilising microwaves and solar energy is a cost-effective way to dry food because microwaves speed up moisture removal, while solar energy provides a low-cost, renewable heat source [38]. This technique can also improve DR because it takes less time to attain the target final moisture level, as solar energy removes exterior moisture and microwaves remove inside moisture [39]. Simultaneously, microwave-vacuum drying preserves heat-sensitive nutrients while improving quality and energy efficiency by combining electromagnetic radiation with low pressure to enable quick dehydration at lower temperatures [40]. By lowering drying temperatures, combining vacuum and microwave drying helps reduce deterioration in organoleptic qualities. This combination boosts the rehydration ratio because it causes less damage to the food’s microstructure than other drying methods [41].

These two configurations exhibit an important difference in the design of combined drying systems. Sequential drying provides more flexibility as each drying can be independently optimised for a specific moisture range. Thus, a relatively inexpensive drying technique can remove the initial free water before a more energy-intensive technique is used during the falling-rate period. This strategy reduces the operating time, cost, and energy consumption of time-consuming drying technologies such as freeze-drying [42]. In contrast, simultaneous systems can strongly intensify heat and mass transfer but commonly require more sophisticated equipment and process control. Therefore, the selection between sequential and simultaneous operation should integrate the evaluation of multiple factors, including the fruit matrix, energy demand, target moisture content, quality requirements, and equipment complexity and operability.

#### 5.1.1. Structural and Physicochemical Transformation Mechanisms

In combined drying techniques, structural and physicochemical transformations result in enhanced microscopic porosity, greater rehydration capability, and decreased colour variation [43]. The improved mass transfer characteristics can also accelerate moisture removal, as demonstrated by IR-HAD, which reduced drying time by 31.25–38.1% compared to conventional HAD. Consequently, both the material’s structural integrity and biological functionality are improved, while operational costs are significantly reduced. For instance, Infrared Hot-Air drying (IR-HAD) has been identified as an efficient drying technique because it ensures the preservation of quality, uniform heat distribution, and precise temperature control [44]. Although infrared heating provides rapid energy transfer, it has limited penetration depth, making its performance dependent on sample thickness and geometry. Furthermore, an increase in drying rate (DR) at higher microwave power has been reported, primarily due to greater energy input, intensified heat and mass transfer, and enhanced piffing effects that promote the formation of a porous structure and higher internal vapour pressure [45]. Additionally, energy balance is used to measure the quality of energy storage, electromagnetic-to-thermal energy conversion, latent heat of evaporation, and conductive and convective heat transport [46]. Through the integration of various energy sources and processes, CDTs provide greater control over the drying process, enabling better drying conditions and higher-quality product [47]. Nevertheless, increasing energy inputs does not necessarily yield proportional improvements in product quality. These trade-offs demonstrate that structural modification during combined drying can be beneficial only within an appropriate operating window.

#### 5.1.2. Modelling, Monitoring, and Control Mechanisms

Recent developments have highlighted the fundamental role of modelling, monitoring, and control mechanisms in food drying, particularly in optimising fruit drying. An intelligent control system regulates temperature, hot-air velocity, and drying time to improve efficiency, while real-time monitoring of physicochemical parameters ensures consistent quality [48]. Furthermore, drying kinetics and machine learning models enable accurate prediction and optimisation of dryer performance [49]. Lastly, the incorporation of sensors facilitates accurate regulation of moisture content, temperature, and humidity [50]. When combined, these strategies improve energy efficiency, product quality, and process accuracy.

These technological advancements are particularly important because achieving high-quality dried fruits requires uniform temperature distribution and consistent airflow within the drying system. Moreover, optimising critical drying parameters, including temperature, drying rate, airflow velocity, drying time, and humidity, is essential for maintaining product quality. This is especially crucial given the sensitivity of fruits to heat, where improper control may lead to phase transitions or over-drying, ultimately resulting in quality degradation [51]. Collectively, these strategies contribute to improved energy efficiency, enhanced product quality, and greater process reliability.

#### 5.1.3. Effective Diffusivity

When analysing, designing, and optimising heat and mass transfer during fruit drying operations, effective diffusivity (D_eff_) is a crucial metric. In the mathematical modelling of drying behaviour, two types of effective diffusivity are typically used, namely temperature-dependent effective diffusivity (TDED) and moisture-dependent effective diffusivity (MDED) [52]. Therefore, it is crucial to determine D_eff_ accurately, as it directly affects the drying model’s reliability and predictive power. Additionally, the drying process is governed by the effective diffusivity, which scales the drying rate. More specifically, increased internal moisture migration and greater mass-transfer efficiency result in a higher drying rate as diffusion efficiency rises [53]. In addition to internal mass transfer, external factors such as heating uniformity and surface moisture dynamics also play a significant role in drying performance. Research indicates that improving heating uniformity and avoiding moisture buildup on the food surface enhances the product’s qualitative and organoleptic properties. Additionally, lowering surface moisture in CDT prolongs shelf life [54]. Furthermore, in previous research, rapid heating and a greater decrease in surface moisture at higher microwave and infrared powers were the reasons for the decrease in activation energy with increasing power [55].

#### 5.1.4. Processing Time

Salehi et al. [56] studied the effects of different IR power (300 W–400 W) and vacuum (5 kPa–25 kPa) on the infrared vacuum drying of lemon slices. The findings indicated that the quickest drying time was achieved with increased vacuum and IR power. Most combined dryers, including microwave-assisted vacuum dryers [57,58], infrared-assisted hot air dryers [59], and ultrasound-assisted hot air dryers [60,61], can reduce the time required for drying and heating by 35–50%. Thus, by avoiding microstructural changes, reducing colour change, maintaining higher levels of nutrients/bioactive compounds, and preserving flavour/aroma. The synergistic effect of combined drying can improve product quality when coupled with an appropriate combination of heating modes. Even though reduced drying time is a common advantage of combined drying, the magnitude of the improvement depends heavily on the combination of technologies and operating conditions. Therefore, time reduction alone should not be the sole criterion for identifying the most suitable combined drying technology, as the criteria should include energy requirements, quality retention, equipment complexity, process controllability, and, particularly, technology scalability.

#### 5.1.5. Drying Kinetics

Fluidised bed (FB) drying has been combined with MW drying to prepare nutmeg mace. A benefit of fluidised bed drying is its improved mass and heat transfer, which makes it well-suited for drying fruits. By accelerating heat generation and raising product temperatures throughout the drying process, the results demonstrate a 60–70% reduction in drying time. A 50% in drying time was achieved by optimising the drying parameters of 48.24 °C and 637.43 W. Compared with convective drying (4.15 h) and sun drying (16 h), the improved procedure (MW-FB drying) also demonstrated a shorter drying time (1.3 h) [62].

A hybrid infrared and heat-pump drying method was used to dry yam tubers. The air velocity was 10 m/s, and the IR power ranged from 500 W to 2000 W. About 700 min and 7.85 kWh of energy were required for HP drying alone to reduce the yams’ moisture content to less than 20%. Combining the heat pump (HP) and infrared (IR) reduced the drying time to 250 min with an energy consumption of 4.39 kWh, representing 44.08% of the baseline energy usage [63].

These quantitative comparisons highlight that the benefits of combined drying arise not purely from adding a second energy source but from strategically addressing the rate-limiting stage of moisture removal. For instance, the substantially reduced drying time observed with microwave-FB drying can be attributed to complementary effects of volumetric microwave heating and efficient convective heat and mass transfer in the fluidised bed. Similarly, the reduction in both drying time and energy demand observed in infrared-assisted heat-pump drying suggests that process intensification can compensate for the relatively slow drying rate associated with low-temperature heat-pump systems. Nevertheless, these findings should be interpreted with caution, as energy savings and reduced drying time reported under laboratory-scale conditions may not translate directly to industrial operations, where equipment efficiency, process control, loading capacity, and auxiliary energy consumption are highly important.

## 6. Thematic Structure and Evolution of Research in Combined Drying Techniques for Fruit Quality

**Figure 3:**
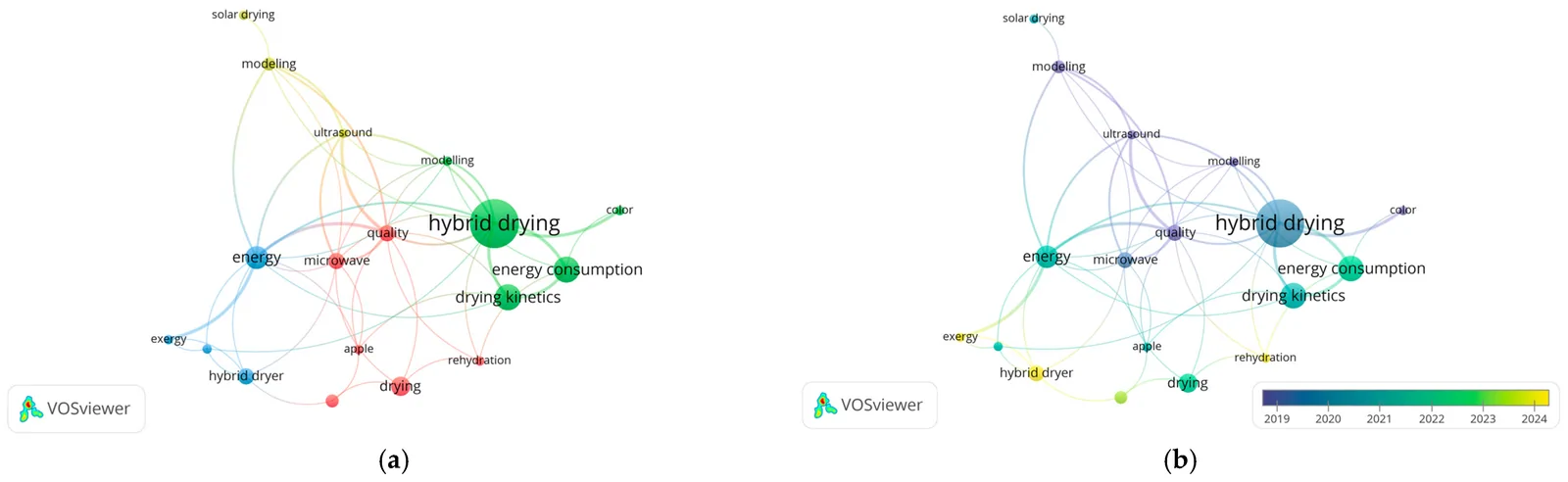
Network visualisation (**a**) and overlay visualisation (**b**) of combined drying techniques for fruit quality using the author’s keywords. Each node represents a keyword, and its size indicates the keyword’s frequency. A larger node indicates a high publication count, while a link between two keywords shows the frequency of their co-occurrence. The thickness of the links between nodes signifies the frequency with which keywords co-occur. In (**a**), different colours indicate distinct clusters and in (**b**), the colour scale exhibits the average publication year of each keyword, where the colour coding for each keyword is defined by the average publication year of all publications with the keyword.

Research clusters and trends of combined drying techniques for fruit quality were mapped using the author’s keywords, as illustrated in Figure 3. The results revealed four interconnected research clusters, highlighting the multidisciplinary and integrative nature of CDT research. The cluster labelled with green colour is centred on hybrid drying, the most prominent and highly connected keyword. Its strong association with drying kinetics, energy consumption, and modelling suggests that research on hybrid drying techniques is primarily focused on process optimisation (energy efficiency) and drying performance evaluation. The second cluster (red) mainly focuses on quality attributes, linked to energy efficiency, highlighting the growing interest in quality-driven optimisation of drying techniques, particularly microwave-related drying methods. This cluster also indicates apple as a model crop for research on hybrid drying techniques. In addition, the research clustering also revealed an energy-oriented research cluster (blue), linking energy, solar dryer, hybrid dryer, and exergy analysis. This cluster reflects a strong emphasis on thermodynamic analysis and the use of renewable energy in CDT research. Lastly, the results showed a research cluster (yellow) focusing on modelling, ultrasound, and solar-assisted drying, which represent emerging approaches to enhance drying efficiency and mechanistic understanding (moisture removal behaviour). Overall, the author’s keyword analysis revealed strong thematic integration among process-, performance-, and quality-oriented research. Furthermore, the network witnessed a shift in CDT research toward optimisation-driven, application-oriented studies. The observed shift may reflect a broader evaluation of modern drying research, which increasingly seeks to improve process efficiency, product quality, and sustainability. The complexity of combined drying systems, with multiple processing variables and their interactions, makes optimisation essential to determine suitable operating conditions.

The overlay visualisation shows the temporal evolution of research on combined drying techniques for fruits (Figure 3b). While early studies of the last decade (2016–2020) focused primarily on modelling, ultrasound, and quality, recent studies (from 2022) have converged on hybrid dryer development, exergy analysis, and energy efficiency, highlighting more applied, efficiency-driven investigations.

## 7. Factors Affecting the Combined Drying Process Efficiency

Combined drying technologies are increasingly used as preservation methods in the fruit industry, as they effectively reduce postharvest losses, preserve the nutritional quality of the dried product, and add value. Moreover, they play a significant role in extending the shelf life of dried fruits by reducing microbial growth. However, their efficacy depends on interactions among numerous factors, including fruit characteristics, operating conditions, and technology-specific parameters (Table 2). Product-related factors (porosity, thickness, and initial moisture content) influence heat and mass transfer, while process parameters (temperature, airflow, and relative humidity) determine the drying environment. In combined drying systems, additional technology-specific parameters, such as microwave power, infrared intensity, vacuum pressure, and the transition point between sequential drying stages, further influence drying kinetics, energy demand, and final product quality. Therefore, it is imperative to have an in-depth understanding of the fundamental factors that affect the drying process.

## 8. Pretreatment-Assisted Drying Versus Combined Drying Technologies

It is important to differentiate between combining two drying mechanisms and integrating a pretreatment with a subsequent drying operation. Pretreatments encompass a wide range of thermal, non-thermal, chemical, and mass-transfer-based interventions applied before the principal drying operation to modify product properties and improve subsequent drying performance. Among these, technologies such as pulsed electric field, ultrasound, and cold plasma primarily modify the structural or physicochemical properties of the fruit matrix before the primary drying operation, rather than serving as independent drying methods. These technologies commonly aim to increase cellular permeability, reduce internal or external mass transfer resistance, and facilitate subsequent moisture removal. Therefore, pretreatment-assisted drying is distinguished here from combined drying technologies, in which two or more drying mechanisms are deliberately integrated as the principal dehydration stages. Nonetheless, some pretreatment technologies, such as osmotic dehydration, can remove a substantial fraction of moisture. In such cases, the distinction is based on the technology’s primary functional role within the overall process.

### 8.1. Pulsed Electric Field (PEF)

Pulsed electric field pretreatment induces electroporation of plant cell membranes, which increases tissue permeability and facilitates moisture transfer during subsequent drying processes [72,73,74]. PEF treatment has been shown to increase effective moisture diffusivity by approximately 1.5–3-fold and reduce drying time compared to untreated samples [75,76]. The structural modifications resulting from PEF can also improve rehydration and colour retention; however, its effects on texture and nutrient retention are product- and treatment-intensity-dependent [77]. Additionally, reductions in subsequent drying time may offset the additional electrical energy required for PEF pretreatment, as demonstrated by convective drying of PEF-pretreated apples [78]. Therefore, the benefit of PEF pretreatment should be assessed based on the net energy consumption of the entire pretreatment-drying process.

### 8.2. Ultrasound Pretreatment (US)

Ultrasound pretreatment enhances mass transfer through cavitation, in which the formation and collapse of microscopic bubbles cause mechanical disruption of plant tissue and create microchannels that facilitate moisture migration during subsequent drying [79,80]. The effectiveness of US depends on parameters such as frequency, power intensity, treatment time, and temperature [81]. Studies have reported shorter drying times following US pretreatment, with the disruption of cellular structures and formation of pores facilitating moisture movement during infrared and vacuum drying [82,83]. US pretreatment can also improve product quality, including β-carotene retention, rehydration capacity, and colour preservation [84].

From an energy perspective, reductions in subsequent drying time may compensate for the electrical energy required for US treatment. Reductions in specific energy consumption of up to 70% have been reported for US-assisted freeze-drying [85], while lower overall energy requirements have been reported for US-assisted convective drying [86]. However, the energy efficiency of the US cannot be generalised across fruits and configurations, as it is highly dependent on treatment intensity, duration, frequency, fruit structure, and subsequent processing methods. Therefore, optimisation is required to balance mass transfer, energy savings, and quality retention [87].

### 8.3. Cold Plasma Pretreatment (CP)

Cold plasma pretreatment is a non-thermal pretreatment in which reactive oxygen and nitrogen species interact with the food surface, resulting in both physicochemical and structural modifications [88,89]. Unlike pulsed electric field and ultrasound, which predominantly modify cellular permeability and internal mass-transfer pathways, CP primarily acts on the fruit surface by etching the cuticle and wax layers and creating microchannels, thereby reducing surface resistance to moisture transfer [90]. In products such as jujube and apple, these structural changes have been shown to increase drying rate and decrease drying time [91,92]. Additionally, CP provides a quality-related benefit by inactivating enzymes, thereby limiting enzymatic browning and other enzyme-mediated quality deterioration during subsequent drying and storage [93]. Enhanced retention of phenolics, flavonoids, and procyanidins, along with reduced hydroxymethylfurfural (HMF) formation, has been observed following CP pretreatment [94,95]. Improved moisture transfer can also lower energy requirements, with reported reductions of approximately 25–46% in specific energy consumption during combined drying [96]. Nevertheless, various factors influence CP effectiveness, including treatment time, plasma-generating gas, voltage, and the product surface.

### 8.4. Osmotic Dehydration Pretreatment (OD)

Osmotic dehydration pretreatment differs from other non-thermal pretreatments in that it directly removes water from fruit tissues before the main drying step via an osmotic pressure gradient established by immersion in a hypertonic solution [97,98]. The extent of water loss is influenced by factors such as solute concentration, temperature, immersion time, and the type of osmotic agent used [99]. This partial dehydration can significantly reduce the subsequent drying load; for instance, OD has been shown to reduce the drying load by 20–70% in vacuum-dried kumquat slices, with 48–68% of water removed within the first three hours of osmotic treatment [100]. OD can also enhance colour preservation [101] and, depending on the osmotic agent, improve quality attributes such as carotenoid retention and water activity [102]. In contrast to PEF, ultrasound, and cold plasma, OD involves both water loss and solute uptake, which can alter the composition, flavour, and texture of the final product [103]. Furthermore, the apparent energy advantage of OD should be evaluated at the process level, as the preparation, concentration, and regeneration of the osmotic solution may significantly contribute to the overall energy requirement [104]. Therefore, OD is particularly advantageous before energy-intensive drying operations owing to its ability to remove substantial moisture prior to drying, although solute uptake and osmotic-medium management remain important considerations for product quality and process sustainability.

Overall, pretreatments that assist drying employ diverse mechanisms (structural modifications of the fruit or reduction in the drying load) before the principal drying stage. Hence, their effectiveness depends on the fruit matrix, the subsequent drying method, the operating conditions, and the targeted quality. However, such process intensification does not constitute a combined drying technology. Therefore, pretreatment should be evaluated across the entire pretreatment-drying chain, considering drying efficiency, energy and resource requirements, quality trade-offs and overall process sustainability.

## 9. Classification and Characteristics of Combined Drying Techniques in Fruit Drying

Numerous scholars have extensively investigated the efficacy of combined drying techniques as a strategic approach to addressing the inherent limitations of the conventional single-drying methods in fruit processing. Empirical findings indicate that integrating multiple drying techniques can enhance the retention of bioactive compounds, reduce specific energy consumption, and lower overall production costs [105,106,107,108]. However, these advantages are not universal and depend on the drying configuration, fruit characteristics, operating conditions, and processing scale. Therefore, combined drying technologies should be evaluated based on their overall quality-energy-time-cost trade-off rather than on individual performance metrics. Consequently, this novel approach has increasingly gained significant attention in recent years.

Furthermore, combined drying techniques can be classified into distinct categories (Table 3), each offering specific advantages based on the operational mechanisms and fruit characteristics.

## 10. Performance Dimensions of CDT in Fruit Drying

### 10.1. Product Quality Preservation

Product quality is a crucial performance dimension for CDT, especially fruit appearance, flavour, texture, and nutrient retention, which highly influence consumer acceptability. CDT has consistently been reported to outperform single drying techniques in retaining quality attributes (colour, aroma, and rehydration) and enhancing techno-functional properties. For instance, microwave drying, while effective in reducing drying time, is often associated with non-uniform results due to the uneven distribution of microwave energy within the drying chamber [121]. Furthermore, excessive microwave exposure can lead to overheating of fruits, undesirable texture changes, and reduced product quality. To address these limitations, microwave drying combined with hot air has been shown to improve the overall quality of the dried product [122].

Conventional hot-air drying (HAD) of fruits and vegetables, especially when conducted at high temperatures or for prolonged periods, can seriously compromise flavour, colour, and nutrients, as well as the rehydration capacity of the dried product [123]. These limitations have contributed to the development and application of alternative combined techniques, as summarised in Table 3.

However, using hot air in freeze vacuum drying improves quality retention (better aroma and rehydration capacity), shortens drying time, and reduces energy consumption by 45.5%. Furthermore, Saxena et al. [124] reported that jackfruit bulb slices dried using freeze vacuum drying–hot air drying produced higher-quality crisps with higher rehydration ratios, shrinkage, instrumental texture, colour values, and sensory scores than hot-air-dried crisps. Similarly, dehydrated bamboo shoot slices from combined freeze-vacuum drying and hot-air drying were superior to those from single hot-air drying in terms of sensory, nutritional, and cellular structure and resulted in 20% lower energy consumption than standalone freeze-drying [125].

Far-infrared (FIR), combined with hot air, has shortened drying times by 35.2% whilst enhancing colour, flavour and active ingredients [126]. FIR heating can liberate covalently bound molecules, increasing bioactivity [127]. Also, when Korean ginseng was dried using a combination of IR and HA dryers, the resulting dried ginseng exhibited higher concentrations of sugars (fructose, glucose, and maltose) and crude saponins than samples dried solely using HA [128].

Microwave-vacuum drying has been widely applied to fruits and vegetables, including apples [129], carrots [130], bananas [131], and tomato slices [132]. These studies reported that microwave-vacuum-dried products exhibited a more porous structure, a softer texture, better rehydration properties, and higher retention of appearance, colour, taste, aroma, and overall quality, while reducing the drying time by 25% [133]. Additionally, notable improvements in lemon colour quality have been observed with far-infrared radiation-pulsed vacuum drying at 60 °C [134]. Likewise, in red peppers, FIR-PVD outperformed infrared-assisted hot-air drying (IR-HAD) by lowering the nonenzymatic browning index, improving rehydration capacity, and maximising red pigment (8.32 ± 0.01 g/kg d.m) and ascorbic acid 28.84–42.44 mg/100 g (d.b) [135]. Overall, it has been demonstrated extensively that strategically combining drying techniques enhances both structural and functional quality while improving process efficiency.

**Figure 4:**
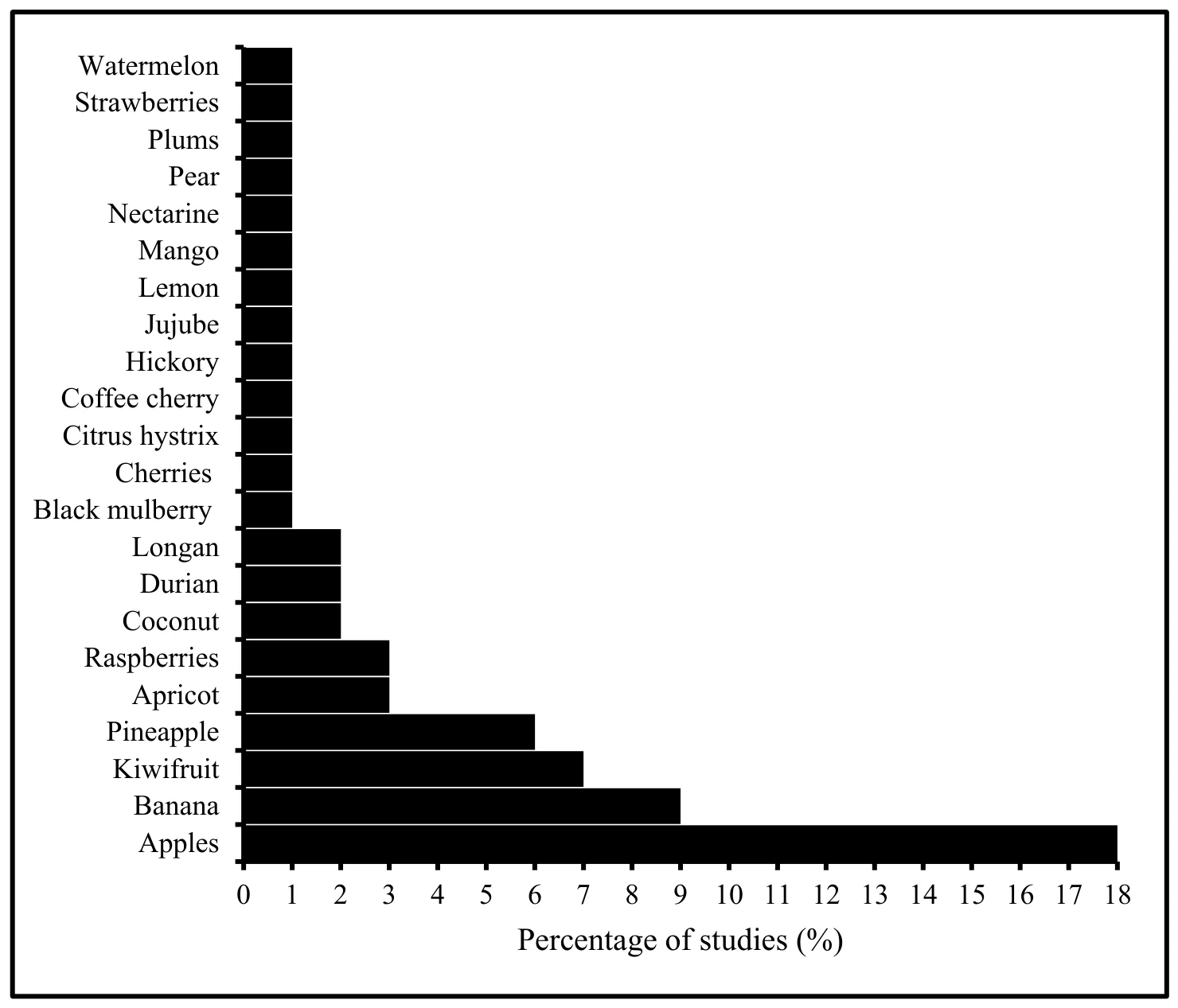
Distribution of fruit commodities investigated in studies on combined drying technologies retrieved from the Scopus database.

Figure 4 illustrates the distribution of fruit commodities investigated in studies on combined drying techniques during the last decade. Thus, approximately 22 different fruits have been investigated, with apple accounting for 21%, emerging as the most frequently studied fruit commodity, followed by banana (11%). These findings underscore the model status of apples in drying research as highlighted earlier in Figure 3a. However, despite the diversity of commodities studied, the absence of pomegranate fruit, a fruit with exceptional nutritional and functional value, represents a critical gap in CDT research. Given its high perishability and nutritional value, this finding highlights the need for future research on CDT for pomegranate that can improve drying efficiency while maintaining its functional quality.

### 10.2. Drying Efficiency

The simplicity and adaptability of hot-air drying have established it as one of the most common drying techniques in the food industry [136]. However, this drying method has the following two critical drawbacks: lengthy drying times and low energy efficiency [137,138]. Microwave drying, by contrast, induces internal heating that accelerates moisture migration to the surface, speeding up the drying process compared to convective methods [139,140]. However, uneven energy distribution in microwave drying can lead to a possible localised overheating. Therefore, combining HA and MW drying techniques can help overcome the drawbacks of individual dryers.

### 10.3. Energy and Cost Considerations

In the context of rising energy costs and climate change, energy demand is a decisive factor in selecting drying technologies. Combined drying techniques have demonstrated significant reductions in energy consumption, resulting in lower operating costs than single methods.

Combining drying techniques (CDTs), such as hot air-microwave (HAD-MW), freeze vacuum air (FVD-AD), microwave-assisted freeze drying (MW-FD), heat pump-freeze drying (HPD-FD), and infrared-assisted vacuum system, has demonstrated significant improvement in both energy efficiency and operational cost reduction compared to single drying methods [141]. Research has shown that combining or optimising processes such as FVD-AD and vacuum freeze-drying can reduce energy consumption by about 34–35%, indicating substantial efficiency gains [142]. Microwave-assisted freeze drying (MV-FD) further decreases energy use due to shortened operational times of system components by 35.63%, while heat pump-assisted freeze drying (HPD-FD) is identified as a more cost-effective and energy-efficient option than conventional freeze drying, benefiting from low temperature operation that minimises thermal degradation and reduces overall energy demand by 35.7% [143,144,145].

Combining hot air with microwave (HA-MW) enhances moisture removal, improves heat and mass transfer, and significantly shortens drying duration, resulting in lower total energy input and production costs [146]. Similarly, hybrid systems such as FIR-assisted pulsed vacuum drying and intermittent microwave vacuum drying further improve drying uniformity, reduce energy consumption, and shorten drying time by enhancing heating control and mass transfer efficiency [147]. Overall, shorter drying cycles and integrated energy systems in CDT directly lower operational and capital costs by minimising equipment run time and energy demand, increasing process efficiency, and reducing waste heat.

### 10.4. Environmental Footprint: Carbon Savings, Renewable Energy Integration

Drying is a crucial operation in the fruit industry, but it continues to face significant hurdles due to its high energy consumption. This high energy consumption frequently significantly impacts the environment, especially when greenhouse gas emissions are involved. Nonetheless, combined drying methods have become a viable means of reducing carbon emissions, particularly when integrated with renewable energy systems, thereby enhancing sustainability and energy efficiency.

Solar-assisted dryers have been reported to reduce CO_2_ emissions by up to 430,714.76 tons annually, reduce excessive reliance on conventional energy sources, improve energy accessibility, and minimise drying space, all of which support environmental conservation efforts [148].

Furthermore, Prosapio et al. [149] conducted a life-cycle assessment (LCA) comparing conventional freeze drying with combined freeze drying and osmotic dehydration for industrial strawberry processing. The findings revealed that the processing stage was the primary contributor to environmental impacts, whereas the agricultural, packaging, and end-of-life-cycle stages had only minor effects. Moreover, the combined method demonstrated lower energy consumption and a 25% reduction in life-cycle carbon footprint. Consequently, CDT achieved higher carbon efficiency and greater sustainability potential than the individual drying method.

## 11. Digitalisation and Industry 4.0: Role of AI and ML, IoT-Enabled Smart Drying Systems

The emergence of digital technologies in the Industry 4.0 context has revolutionised drying processes, enabling intelligent, adaptive systems. Digitalisation can advance combined drying systems at four interconnected levels: process monitoring, prediction, optimisation, and adaptive control. Real-time sensors and imaging systems can monitor moisture, temperature, energy consumption, and changes in quality, while machine learning can predict or optimise drying endpoints and product quality. The increasing integration of Artificial Intelligence (AI), the Internet of Things (IoT), and machine learning (ML) into modern drying processes enhances drying efficiency, reduces energy consumption, and improves product quality. AI-integrated control and modelling systems enable real-time monitoring of material behaviour under various drying conditions, allowing adjustment of key drying parameters such as temperature and airflow. This leads to higher quality results, more consistent drying, and reduced energy consumption [150].

Machine learning algorithms are trained on sensor feedback to predict drying efficiency (kinetics), temperature uniformity, and product endpoint quality in real time [151]. In addition, real-time images of dried samples have been captured using computer vision to analyse colour changes in fruits and vegetables during drying [152]. These algorithms enable the prediction of drying models and the control of drying parameters, thereby supporting more efficient drying operations and potentially reducing operating costs; the CPV/TSD system achieved an overall thermal energy efficiency of 20% [153].

Furthermore, electronic noses, an olfactory technology, have demonstrated their potential to assess changes in volatile compounds over the drying period. For instance, Makarichian et al. [154] successfully distinguished the aroma profiles of garlic produced by various drying methods, while Zhang et al. [155] examined changes in volatile compounds in coffee beans during drying using electronic-nose sensors. Therefore, leveraging such digital tools in a combined drying technology for fruit drying can significantly support smart drying, thereby improving both dried fruit quality and the sustainability of the drying process.

However, as most intelligent drying approaches remain at laboratory or proof-of-concept level, the industrial implementation of digital tools in combined drying technologies is constrained by limited data availability, model transferability, and sensor reliability under harsh processing conditions.

## 12. Alignment with Sustainable Development Goals (SDGs) of Combined Drying Systems

Combined drying systems can support several Sustainable Development Goals (SDGs) by improving food preservation, integrating renewable energy, enhancing food security, alleviating poverty, reducing environmental impacts, and promoting sustainable agriculture [156,157]. However, their contributions should be assessed based on measurable technological and environmental outcomes. Hence, the available literature indicates that most contributions are related to SDGs 2, 7, 9, 12, and 13.

SDG 2 Zero Hunger: giving small-scale producers access to cutting-edge CDT enhances rural productivity and income-generating opportunities [158]. In addition, improving efficiency allows long-term food preservation, ensuring food availability during drought seasons [159]. This contributes to food security by extending the shelf life of perishable fruits and reducing postharvest losses. However, its contribution depends on the practical implementation of the technologies.

SDG 7 Affordable and Clean Energy: The contribution of combined drying to this SDG is highly relevant, particularly when the drying system integrates renewable energy sources. Continuous dryers can be improved through biomass operation, heat pumps, geothermal heat exchangers, and electricity [160,161]. Thermal energy storage enhances drying efficiency and extends operation by up to five hours when exposed to no direct sunlight [162]. Biomass and biogas offer renewable, carbon-neutral and circular energy solutions that reduce greenhouse gas emissions [163,164].

SDG 9 Industry, Innovation and Infrastructure: CDT enhances energy efficiency, supports scalable applications for both small and large industries, and improves product quality while maintaining a consistent drying environment [165,166]. These innovative drying techniques promote sustainable industrial growth and align with SDG9.

SDG 12 Responsible Consumption: CDT supports responsible consumption by producing nutritious, high-quality dried fruits with lower energy usage. These advanced drying techniques preserve nutrients, reduce environmental impact, and promote global sustainability [167].

SDG 13 Climate Action: According to [168], nonthermal technologies improve food safety and sustainability while reducing greenhouse gas emissions, and [169] develops a PV/T GHD system that reduces moisture content from 80% to 11% wb in 20 h, maintaining mint natural colour and nutrients. The system achieved 34.2% efficiency and reduced CO_2_ emissions by 140.97 tons, contributing significantly to climate action. However, evidence directly quantifying the carbon footprint and life-cycle impacts of combined fruit-drying systems remains scarce, underscoring the need for a comprehensive life-cycle assessment.

## 13. Challenges, Research Gap and Future Directions

Despite growing interest in combined drying techniques to enhance fruit quality, the literature lacks a comprehensive analysis of policy, economic, and market factors, including incentives, renewable energy policies, and demand for clean-label, sustainable dried fruits. Furthermore, information on translating combined drying technologies from laboratory scale to industrial applications remains scarce in the literature. Although numerous studies have reported improvements in drying kinetics, energy efficiency, and product quality, empirical evidence regarding industrial applications of combined drying technologies remains uncertain. Yet industrial adoption extends beyond factors influencing technical performance to encompass capital and operating expenditures (CAPEX and OPEX), process controllability, maintenance requirements, technological readiness level (TRL), and compatibility with real-world production lines. Hence, technical performance metrics (drying time, energy consumption) cannot be considered in isolation to reliably assess the sustainability of the combined drying configurations. This knowledge gap is particularly crucial, as larger processing capacities introduce challenges related to heat and mass transfer uniformity, product loading capacity, and energy distribution. Consequently, the industrial feasibility of emerging laboratory-scale combined drying technologies remains uncertain, as the maturity of the individual drying technologies does not necessarily translate to equivalent maturity when combined because their integration introduces additional requirements for process control, equipment compatibility, safety, maintenance, and energy management.

Therefore, future research should move beyond optimising drying performance and quality retention to pilot- and large industrial-scale validation. Hence, future research on combined drying technologies should integrate processing capacity, maintenance requirements, process controllability, economic indicators (CAPEX, OPEX, payback period and Return On Investment), and environmental impact assessments. These indicators will enable a reliable assessment of the technology readiness and commercialisation potential of combined drying technologies, promoting their adaptation.

In parallel, significant attention should be given to the development of continuous, modular, combined drying systems, the integration of renewable energy sources, and smart process control strategies.

## 14. Conclusions

This critical review demonstrates that combined drying techniques (CDTs) have been increasingly studied in fruit drying and can address certain limitations associated with single drying procedures. Depending on the drying configuration, fruit characteristics, and processing conditions, CDTs can increase drying efficiency, improve retention of bioactive compounds, and enhance the functional and nutritional qualities of dried fruits. In addition, they can lower production costs, minimise energy use, and reduce greenhouse gas emissions. However, the magnitude and nature of these benefits vary substantially across associated drying technologies and fruit matrices, and improvements in one performance indicator may entail trade-offs in others. Although these technologies strongly support several Sustainable Development Goals (SDGs), their broad adoption is still hampered by several obstacles, including high upfront costs, insufficient legislative backing, and technical challenges that prevent large-scale deployment.

## Figures and Tables

**Table 1:** Categories of combined drying techniques for fruits.

Categories	Examples	References
Combined drying	Microwaves + hot air Hot air + freeze drying	[24] [26]
Hybrid drying	Solar + vacuum drying Solar + electric	[27]

**Table 2:** Factors that affect the drying process.

Factor Category	Factors	Effect	Reference
Product-related	Food material	Products may be overcooked or undercooked during the drying process if certain drying parameters, dryer type, and product attributes are not appropriately considered.	[64]
Technology-related	Dryer specifications	For various food variety, the drying process is greatly influenced by the dryer selection. The type of food, drying performance, energy source, and drying efficiency all play a role in choosing the right dryer.	[65]
Process-related	Temperature	While high temperatures might shorten drying times, they can also cause nutritional changes, nutrient degradation, off-flavour, browning reactions, and decreased microbial activity.	[66]
Process-related	Airflow speed	The drying unit must have optimal drying conditions and uniform airflow distribution to improve the quality of dried fruits and achieve an efficient drying process with the right drier.	[67]
Process-related	Relative humidity	When drying at a consistent rate, high relative humidity may promote heat transfer and speed up drying. Low relative humidity, on the other hand, promotes moisture diffusion throughout the period of declining drying rate.	[68]
Product-related	Moisture content	Fruits and vegetables with high moisture content tend to prolong the drying time, resulting in high energy consumption, while low moisture-content fruits dry faster, conserving energy.	[69]
Product-related	Product porosity	One of the most important factors in regulating heat and mass fluxes is porosity.	[70]
Product-related	Layer thickness	It was discovered that in every instance, the drying rate dropped as thickness increased.	[71]

**Table 3:** Classification of combined drying techniques.

Configuration	Combined Drying Technology	Complementary Mechanisms	Key Advantages/Reported Performance	Limitations/Challenges	Fruit Applications	References
Simultaneous	Microwave + vacuum (MW-VD)	Microwave uses electromagnetic waves to create volumetric heating and promote internal vapour-pressure development, while the vacuum reduces oxidation and heat damage, preserving delicate nutrients and improving heat transfer and evaporation.	Quick drying; reduced thermal and oxidative exposure; retain heat-sensitive and bioactive compounds.	High equipment and vacuum system costs; uneven distribution of microwave heating potential spots in thick or heterogeneous materials.	Saskatoon berry, blueberries, banana	[107,108,109,110]
Simultaneous	Hot air + electrohydrodynamic (HAD-EHD)	Convective heating promotes evaporation (hot-air drying), while the EHD-produced ionic wind enhances the surface mass transfer and moisture removal with minimal additional energy input.	Improved moisture removal and drying rate, reduced shrinkage, and enhanced rehydration and colour. In addition, it has been reported that integrating EHD -HAD reduced by 16% and 17% drying time and energy demand, respectively, and increased the rehydration rate by 14%.	Excessive airflow may reduce/suppress the ionic wind effect; increased product loading can alter airflow and the electric field distribution, which may reduce drying uniformity.	Banana, peach	[111,112,113]
Simultaneous or Sequential	Electrohydro-dynamic + freeze drying (EHD-FD)	The integration of electrohydrodynamic with freeze drying removes moisture by sublimation, while high-voltage electric field (EHD) produces an ionic wind that enhances vapour and mass transfer during sublimation.	Approximately 15% reduction in processing time compared to conventional freeze drying; reduced structural shrinkage.	High capital expenditure (CAPEX) and high voltage requirements; limited information on small-scale farming sector and industrial scale up.	Guava, banana, apple	[114,115]
Sequential	Hot-air drying + freeze drying (HAD-FD)	Hot-air drying substantially removes bulk-free water through convective heat and mass transfer, while the subsequent freeze drying completes dehydration by sublimation under low temperature and vacuum pressure.	Reported reductions in drying time by 39–50% and in energy consumption/processing costs by 40–56%, while maintaining dried product quality.	Strongly depends on the transition point between HAD and FD, limited techno-economic and pilot-/industrial-scale validation.	Kiwifruit, mango	[116,117]
Simultaneous	Infrared + hot-air drying (IR-HAD)	Convective heat promotes surface evaporation and moisture removal, while IR provides radiative surface heating, which raises the temperature and moisture gradient.	Reduced shrinkage and activation energy; increased drying rate, reported reductions of 34.5% in drying time and 38.6% in specific energy use, with low greenhouse gas emissions.	Its poor penetration depth can reduce the effectiveness, particularly in thick fruits; this technology is highly dependent on IR intensity, distance, wavelength, and product geometry.	Mango, blackberry, white mulberry	[118,119,120]

## Data Availability

No new data were created or analysed in this study. Data sharing is not applicable to this article.

## Abbreviations

The following abbreviations are used in this manuscript:

CDTs	Combined drying techniques
DRs	Drying rates
SDGs	Sustainable development goals
LCA	Life-cycle assessment
TDED	Temperature-dependent effective diffusivity
MDED	Moisture-dependent effective diffusivity
D_eff_	Effective diffusivity
IR-HAD	Infrared-hot-air drying
FB	Fluidized bed
MW	Microwave
FD	Freeze drying
MW-FB	Microwave freeze drying
FIR	Far-infrared
FIR-PVD	Far-infrared radiation—Pulsed vacuum drying
HAD-MW	Hot air-microwave
FVD-AD	Freeze vacuum air drying
